# The Multifaceted Role of Irisin in Neurological Disorders: A Systematic Review Integrating Preclinical Evidence with Clinical Observations

**DOI:** 10.3390/neurolint18010015

**Published:** 2026-01-09

**Authors:** Foad Alzoughool, Loai Alanagreh, Yousef Aljawarneh, Haitham Zraigat, Mohammad Alzghool

**Affiliations:** 1 Department of Medical Laboratory Sciences, Faculty of Applied Medical Sciences, The Hashemite University, Zarqa P.O. Box 13115, Jordan; loai-alanagreh@hu.edu.jo; 2 Department of Nursing, Faculty of Health Sciences, Higher Colleges of Technology, Abu Dhabi P.O. Box 25026, United Arab Emirates; yaljawarneh@hct.ac.ae (Y.A.); hzraigat@hct.ac.ae (H.Z.); 3 Faculty of Medicine, Wuhan University, Wuhan 430072, China

**Keywords:** irisin, neurological diseases, Parkinson’s disease (PD), Alzheimer’s disease (AD)

## Abstract

**Background:** Irisin, an exercise-induced myokine, has emerged as a potent neuroprotective factor, though a systematic synthesis of its role across neurological disorders is lacking. This review systematically evaluates clinical and preclinical evidence on irisin’s association with neurological diseases and its underlying mechanisms. **Methods:** Following PRISMA 2020 guidelines, a systematic search of PubMed/MEDLINE, Scopus, Web of Science, Embase, and Cochrane Library was conducted. The review protocol was prospectively registered in PROSPERO. Twenty-one studies were included, comprising predominantly preclinical evidence (n = 14), alongside clinical observational studies (n = 6), and a single randomized controlled trial (RCT) investigating irisin in cerebrovascular diseases, Parkinson’s disease (PD), Alzheimer’s disease (AD), and other neurological conditions. Eligible studies were original English-language research on irisin or FNDC5 and their neuroprotective effects, excluding reviews and studies without direct neuronal outcomes. Risk of bias was independently assessed using SYRCLE, the Newcastle–Ottawa Scale, and RoB 2, where disagreements between reviewers were resolved through discussion and consensus. Results were synthesized narratively, integrating mechanistic, pre-clinical, and clinical evidence to highlight consistent neuroprotective patterns of irisin across disease categories. **Results:** Clinical studies consistently demonstrated that reduced circulating irisin levels predict poorer outcomes. Lower serum irisin was associated with worse functional recovery and post-stroke depression after ischemic stroke, while decreased plasma irisin in PD correlated with greater motor severity, higher α-synuclein, and reduced dopamine uptake. In AD, cerebrospinal fluid irisin levels were significantly correlated with global cognitive efficiency and specific domain performance, and correlation analyses within studies suggested a closer association with amyloid-β pathology than with markers of general neurodegeneration. However, diagnostic accuracy metrics (e.g., AUC, sensitivity, specificity) for irisin as a standalone biomarker are not yet established. Preclinical findings revealed that irisin exerts neuroprotection through multiple mechanisms: modulating microglial polarization from pro-inflammatory M1 to anti-inflammatory M2 phenotype, suppressing NLRP3 inflammasome activation, enhancing autophagy, activating integrin αVβ5/AMPK/SIRT1 signaling, improving mitochondrial function, and reducing neuronal apoptosis. Irisin administration improved outcomes across models of stroke, PD, AD, postoperative cognitive dysfunction, and epilepsy. **Conclusions:** Irisin represents a critical mediator linking exercise to brain health, with consistent neuroprotective effects across diverse neurological conditions. Its dual ability to combat neuroinflammation and directly protect neurons, demonstrated in preclinical models, positions it as a promising therapeutic candidate for future investigation. Future research must prioritize the resolution of fundamental methodological challenges in irisin measurement, alongside investigating pharmacokinetics and sex-specific effects, to advance irisin toward rigorous clinical evaluation.

## 1. Introduction

Irisin, a peptide hormone released into circulation following cleavage of the transmembrane protein fibronectin type III domain-containing protein 5 (FNDC5), was first identified as a key mediator of the beneficial effects of exercise on metabolism [1,2]. Recently, growing evidence has highlighted its critical role in the nervous system, where it acts as a neurotrophic and neuroprotective factor. One of the primary mechanisms by which irisin exerts these effects is through the regulation of brain-derived neurotrophic factor (BDNF), a central modulator of synaptic plasticity and cognitive processes. Wrann et al. (2013) demonstrated that exercise upregulates hippocampal BDNF expression via a PGC-1α/FNDC5/irisin pathway, linking physical activity to enhanced memory and learning [2].

Beyond synaptic modulation, irisin confers cellular protection against oxidative stress and apoptosis. In hippocampal neurons, irisin activates intracellular signaling cascades such as Akt and ERK1/2, which stabilize mitochondrial function and reduce amyloid-β-induced neurotoxicity, suggesting a role in Alzheimer’s disease pathology [3]. Similarly, in Parkinson’s disease models, irisin preserved dopaminergic neurons by preventing mitochondrial dysfunction and attenuating neuronal apoptosis, thereby improving motor outcomes [4]. These findings underscore irisin’s capacity to counteract hallmark mechanisms of neurodegeneration.

Irisin also modulates neuroinflammatory processes, which are increasingly recognized as critical contributors to neuronal injury. In a murine model of intracerebral hemorrhage, administration of irisin attenuated microglial activation, reduced levels of pro-inflammatory cytokines (IL-1β, TNF-α), and limited neuronal apoptosis through integrin αVβ5/AMPK signaling [5]. Consistent with these findings, irisin administration in ischemic stroke models enhanced BDNF levels, improved neuronal survival, and promoted functional recovery [6]. Taken together, these data suggest that irisin provides a multi-faceted defense against neuronal injury by integrating neurotrophic support, anti-apoptotic signaling, mitochondrial preservation, and anti-inflammatory modulation.

Given the broad spectrum of protective effects, irisin is increasingly recognized as a promising therapeutic target for neurodegenerative disorders such as Alzheimer’s and Parkinson’s disease, as well as acute cerebrovascular insults like stroke and intracerebral hemorrhage. Collectively, these findings position irisin as a promising mediator linking exercise to neuronal resilience in both neurodegenerative and cerebrovascular disorders. However, despite an expanding body of preclinical and early clinical research, the evidence remains fragmented across disease models, experimental settings, and study designs. A systematic synthesis of the literature is therefore warranted to critically evaluate the strength of existing data, identify knowledge gaps, and determine the translational potential of irisin as a therapeutic or preventive agent in neurological disease. The objectives of this systematic review are threefold: first, to summarize the clinical evidence linking irisin levels in serum, plasma, and cerebrospinal fluid to disease risk, progression, and functional outcomes in human neurological disorders such as stroke, Parkinson’s disease, and Alzheimer’s disease; second, to synthesize pre-clinical findings that elucidate the fundamental neuroprotective mechanisms of irisin, with a specific focus on its roles in modulating neuroinflammation, apoptosis, autophagy, and synaptic plasticity; and third, to evaluate the emerging evidence for the therapeutic potential of irisin administration—either directly or through exercise-mediated pathways across diverse experimental models of brain injury and neurodegeneration, thereby identifying critical gaps and informing future translational research.

## 2. Materials and Methods

### 2.1. Protocol and Registration

This systematic review adhered to the PRISMA 2020 guidelines (Supplementary Files S1 and S2). The review protocol was prospectively registered in PROSPERO (International Prospective Register of Systematic Reviews) prior to data extraction and is available at https://www.crd.york.ac.uk/PROSPERO/view/CRD420251168867 (accessed on 5 December 2025).

### 2.2. Eligibility Criteria

Eligible studies included original experimental research (in vitro and in vivo), clinical observational or interventional studies, and randomized controlled trials that assessed the effects of irisin administration, FNDC5 modulation, or exercise-induced irisin on central nervous system outcomes. Studies involving neuronal cell lines, animal models of neurological disease, or human participants (healthy or with neurological conditions) were included. Neuroprotective outcomes of interest encompassed measures such as neuronal survival, apoptosis, oxidative stress, neuroinflammation, neurogenesis, synaptic plasticity, and cognitive or functional performance. Only English-language publications were considered, with no restrictions on publication year. Review articles, editorials, commentaries, conference abstracts lacking primary data, and studies failing to report direct neuronal or neuroprotective outcomes were excluded.

### 2.3. Information Sources and Search Strategy

This systematic review adhered to the PRISMA 2020 guidelines (Supplementary Files S1 and S2). A systematic search was conducted across PubMed/MEDLINE, Scopus, Web of Science, Embase, and the Cochrane Library. Reference lists of included studies were screened manually for additional relevant publications. The search strategy combined terms and free-text keywords such as “irisin” or “FNDC5” with neurological terms (e.g., “neuroprotection,” “neuron,” “brain,” “cognitive function”) and disease-related terms (e.g., “Alzheimer’s disease,” “Parkinson’s disease,” “stroke,” “depression”). A pilot search was first performed in PubMed, and refinements were made in consultation with an academic librarian.

### 2.4. Study Selection

All retrieved records were imported into a reference management system (EndNote), where duplicates were removed. Two independent reviewers screened titles and abstracts according to the eligibility criteria, followed by full-text assessment of potentially relevant studies. Any disagreements were resolved through discussion or consultation with a third reviewer.

### 2.5. Data Extraction

Data from included studies were extracted using a standardized form. Extracted variables included study identifiers (authors, year, country, and funding), study design (in vitro, in vivo, human), model or population characteristics, intervention details (dose, route, duration, or exercise protocol), assessed neuroprotective outcomes, and key findings or conclusions.

### 2.6. Risk of Bias Assessment

Risk of bias was independently assessed by two reviewers using validated tools appropriate to each study design. Observational clinical studies were evaluated using the Newcastle–Ottawa Scale (NOS), randomized controlled trials were assessed using the Cochrane Risk of Bias 2 (RoB 2) tool, and preclinical animal studies were appraised using the SYRCLE risk of bias tool. In vitro studies were assessed using adapted Office of Health Assessment and Translation (OHAT) risk-of-bias domains. Disagreements between reviewers were resolved through discussion and consensus. Overall risk-of-bias judgments were derived according to predefined, tool-specific criteria, as detailed in the legends of Supplementary Tables S1–S4.

## 3. Results

### 3.1. Study Selection

**Figure 1:**
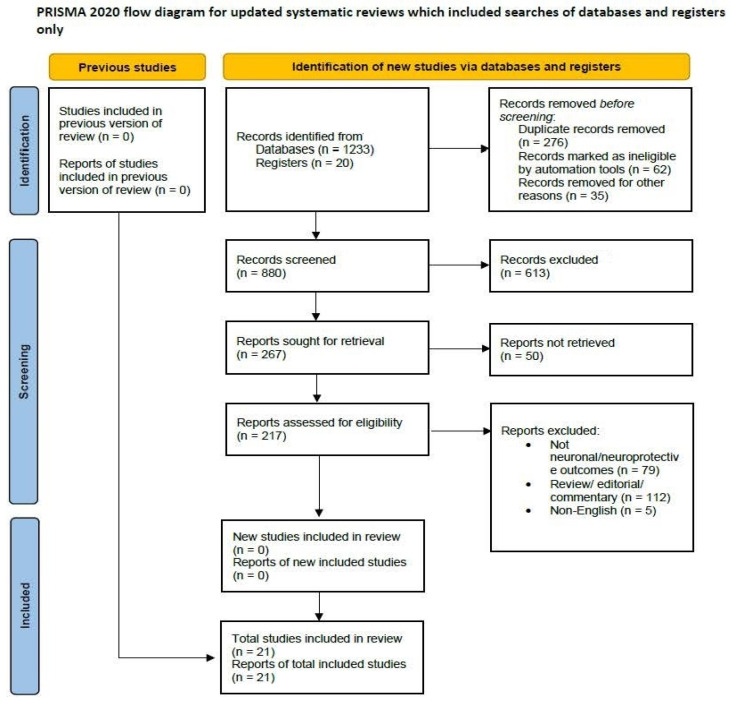
PRISMA flow diagram for studies on irisin and neuroprotection.

The initial systematic literature search across databases (PubMed, Scopus, Web of Science, Embase, Cochrane Library) and other sources yielded 1253 records. After the removal of 373 duplicates, 880 unique records remained. Based on title and abstract screening, 663 records were excluded, leaving 217 full-text articles for eligibility assessment. Of these, 196 articles were excluded with reasons, primarily for not focusing on neuronal or neuroprotective outcomes, being review articles, editorials, or commentaries, or being published in a non-English language. Ultimately, 21 studies met all inclusion criteria and were selected for qualitative synthesis in this systematic review. The study selection process is detailed in the PRISMA flow diagram (Figure 1).

### 3.2. Study Characteristics

The 21 included studies, published between 2018 and 2025, encompassed a range of study designs investigating the role of irisin in various neurological conditions. The corpus included pre-clinical studies (n = 14) utilizing in vivo animal models and/or in vitro cell cultures, clinical observational studies (n = 6) comprising prospective cohorts and case–control designs, and one Randomized Controlled Trial (RCT). The investigated conditions covered a broad spectrum of disorders, including cerebrovascular diseases (e.g., stroke), neurodegenerative diseases (e.g., Parkinson’s and Alzheimer’s disease), and other states such as postoperative cognitive dysfunction and neuroinflammation. Sample sizes varied considerably, from small experimental groups in pre-clinical work to human cohorts of over 1200 participants (Table 1 and Table 2).

### 3.3. Synthesis of Results

In patients with cerebrovascular diseases, lower serum irisin levels were consistently associated with poorer outcomes, predicting both poor early functional recovery and post-stroke depression after ischemic stroke. It is critical to note that these clinical studies are observational; therefore, these associations do not establish causality. Reduced irisin levels could be a consequence of the disease state—such as systemic inflammation, physical inactivity, or muscle atrophy following stroke—rather than a causative driver of poor outcomes. Among the included studies, one randomized controlled trial (RCT) provided preliminary interventional data [20]. This trial investigated staged acupuncture in ischemic stroke patients and reported an increase in serum irisin levels that correlated with improved neurological function, balance, and reduced spasticity. It is crucial to note that this study is an outlier in design and intervention, as it examined a specific physical therapy (acupuncture) rather than direct irisin administration. Its findings should therefore be interpreted with caution and not be conflated with the biomarker associations from observational studies. It does, however, provide tentative, indirect evidence that interventions which may elevate irisin could be beneficial. Mechanistically, in models of intracerebral hemorrhage, irisin treatment improved outcomes by reducing brain edema, neuroinflammation, and neuronal apoptosis via the integrin αVβ5/AMPK signaling pathway.

A strong consensus emerged on the protective role of irisin in Parkinson’s disease (PD). Pre-clinical studies demonstrated that irisin reduces pathologic α-synuclein, attenuates neuroinflammation, and protects neurons through pathways involving autophagy and AMPK/SIRT1. Corroborating these findings, observational clinical studies found that PD patients have significantly lower plasma irisin levels, which correlate with greater disease severity, higher α-synuclein, and reduced dopamine uptake. Again, this correlation cannot be interpreted as causation. The observed reduction in irisin may be secondary to PD pathology, including reduced physical activity, loss of muscle mass, or systemic metabolic changes associated with disease progression. Furthermore, the cognitive and motor benefits of exercise in a PD model were shown to be mediated by the upregulation of irisin.

The relationship between irisin and Alzheimer’s disease (AD) pathology was also extensively investigated. Pre-clinical research established that irisin, whether induced by exercise or administered directly, rescues synaptic plasticity and memory deficits in AD models, with peripheral irisin capable of crossing the blood–brain barrier. In human clinical cohorts, cerebrospinal fluid (CSF) irisin levels were significantly correlated with global cognitive efficiency and performance across multiple cognitive domains. Reduced CSF irisin was correlated with AD progression, with analyses suggesting a closer link to amyloid-β pathology than to general neurodegeneration [22,23,24]. It is crucial to note that the included studies reported correlation coefficients and comparative associations but did not provide formal diagnostic test accuracy data (e.g., AUC, sensitivity, specificity). Therefore, while these findings are promising and suggest irisin merits investigation as a biomarker, they do not yet establish its diagnostic or prognostic validity. The neuroprotective effects of irisin extended to other conditions, including postoperative cognitive dysfunction (POCD), epilepsy, and LPS-induced neuroinflammation. Across these models, irisin consistently ameliorated cognitive impairment, neuronal damage, and inflammatory responses. The key mechanisms identified included the modulation of microglial polarization from a pro-inflammatory M1 to an anti-inflammatory M2 state, suppression of the NLRP3 inflammasome pathway, and the enhancement of mitochondrial function and antioxidant defenses.

### 3.4. Risk of Bias Across Studies

Overall, observational clinical studies demonstrated moderate to high methodological quality based on NOS assessments, with most studies showing adequate participant selection and outcome assessment. Studies with limited control for confounding variables were conservatively rated as moderate quality. The single randomized controlled trial exhibited low risk of bias across most RoB 2 domains. Preclinical animal studies showed predominantly moderate risk of bias, mainly due to insufficient reporting of randomization and blinding procedures. In vitro studies demonstrated low risk of bias, reflecting well-characterized exposures and outcome measurements. Detailed risk-of-bias assessments for individual studies are provided in Supplementary Tables S1–S4.

## 4. Discussion

A critical consideration when interpreting the evidence synthesized in this review is the distinct nature and weight of the available data. The robust and consistent neuroprotective effects of irisin are most firmly established in preclinical models, which provide mechanistic depth and demonstrate therapeutic efficacy upon intervention. In contrast, the clinical evidence remains predominantly observational, revealing important associations between circulating or CSF irisin levels and disease diagnosis, progression, and outcomes. Only one RCT was identified, highlighting a significant gap in high-level interventional evidence in humans. The following discussion therefore integrates these lines of evidence with the understanding that the therapeutic potential of irisin is strongly supported by preclinical science, while its clinical application is a promising prospect awaiting confirmation through rigorous trials. The collective findings underscore a consistent theme: irisin signaling is compromised in neurological diseases, and its restoration, either through physiological means like exercise or via direct administration, confers significant neuroprotection, mitigates neuroinflammation, and improves functional outcomes.

A key and necessary distinction must be made when interpreting the clinical evidence. The most consistent human data on irisin and neurological diseases come from observational cohort studies. In acute ischemic stroke (AIS), low serum irisin levels are a powerful predictor of poor short-term functional recovery [18] and the development of post-stroke depression [19], independent of other variables. Similarly, in Parkinson’s disease (PD), plasma irisin levels are associated with greater motor severity and pathological α-synuclein burden [21]. A significant and plausible alternative explanation is that the disease state itself leads to lower irisin levels. For instance, in stroke and neurodegenerative diseases, factors such as systemic inflammation, prolonged immobility, sarcopenia, and overall metabolic dysfunction—all hallmarks of these conditions—could directly suppress the production or release of irisin from muscle tissue. Therefore, while the correlation is robust and mechanistically supported by preclinical studies, it remains unclear from human data whether low irisin is a contributing cause of neurological dysfunction or primarily a biomarker of the diseased state and its systemic consequences.

The biological interpretation of irisin levels critically depends on the compartment measured. Cerebrospinal fluid (CSF) irisin likely reflects activity within the brain parenchyma, serving as a direct biomarker of central nervous system integrity, as evidenced by its strong correlation with Alzheimer’s disease pathology and cognitive decline. In contrast, peripheral (serum/plasma) irisin, primarily a myokine, may function as an integrative indicator of systemic and muscular health, with its reduction in conditions like stroke and Parkinson’s disease often mirroring systemic consequences such as sarcopenia, inflammation, and inactivity. This compartmental distinction clarifies that CSF irisin is more relevant to intrinsic brain pathology and targeted therapies, while peripheral irisin is valuable for assessing systemic health and the benefits of lifestyle interventions, underscoring the need for future research to specify biofluid context.

In the context of Alzheimer’s disease (AD), the data reveal a more complex picture within the central nervous system. While initial studies focused on serum, recent rigorous analyses of cerebrospinal fluid (CSF) have found that reduced CSF irisin levels are strongly associated with core AD biomarkers (Aβ pathology), global cognitive efficiency (MMSE), and deficits in specific cognitive domains like memory and executive function [19,20,21]. This consistent inverse relationship between irisin levels and disease severity across stroke, PD, and AD highlights its potential as a valuable biomarker for neurological dysfunction.

The therapeutic mechanisms of irisin, extensively elucidated in preclinical models, are multifaceted and converge on two fundamental pathological processes: neuroinflammation and synaptic dysfunction. A key and recurrent finding across numerous studies is irisin’s potent ability to modulate microglia, the brain’s resident immune cells. It does not cause broad immunosuppression but rather induces a phenotypic “reprogramming,” suppressing the pro-inflammatory, cytotoxic M1 state (characterized by high levels of IL-1β, IL-6, TNF-α, iNOS, and CD86) and promoting an anti-inflammatory, reparative M2 state [13,17]. This effect is mediated through the inhibition of key pro-inflammatory pathways, most notably NF-κB [13,17] and the NLRP3 inflammasome [8,13], and the activation of STAT6 signaling [14]. By quenching this neuroinflammatory fire, irisin creates a microenvironment conducive to neuronal survival. This is further supported by its activation of cell-survival pathways like integrin αVβ5/AMPK/SIRT1 [5,9,15], which enhances mitochondrial function [11], reduces oxidative stress [16], and inhibits neuronal apoptosis [5]. Furthermore, irisin directly benefits neurons by rescuing synaptic plasticity and memory deficits in AD models [3,11,12], protecting against excitotoxicity by restoring the glutamate/GABA balance [16], and promoting the autophagy-dependent clearance of pathological proteins like α-synuclein in PD [4,7,8].

The translation of these mechanisms into functional benefits is evident across disease models. In models of postoperative cognitive dysfunction (POCD), irisin pretreatment prevented neuroinflammation, synaptic loss, and cognitive impairment [17]. In intracerebral hemorrhage (ICH) and ischemic stroke models, it improved neurological function, reduced brain edema, and mitigated apoptosis [5,20]. A single RCT by Chen et al. [20] stands apart from the observational corpus. This study reported that staged acupuncture in ischemic stroke patients increased serum irisin levels and correlated this increase with clinical improvement. The intervention was acupuncture, not direct irisin administration, and the rise in irisin was a measured correlation, not a manipulated variable. Therefore, while this study offers intriguing, preliminary evidence that a therapeutic intervention that may modulate irisin levels could have functional benefits, it does not constitute direct proof of irisin’s therapeutic efficacy. It highlights a potential pathway (increased irisin) through which a complex intervention like acupuncture might exert part of its effect, but this requires validation in studies specifically designed to test irisin as a therapeutic agent. In PD models, it ameliorated motor deficits and protected dopaminergic neurons [4,7,11], and in AD models, it reversed synaptic and memory deficits [11,12]. Notably, irisin appears to be a central mediator of exercise-induced neuroprotection, as blocking its signaling abrogates the beneficial effects of exercise on mitochondrial function and apoptosis in a PD model [11].

Despite the highly promising evidence, several challenges and unanswered questions remain. A significant issue is the limited reproducibility of irisin detection assays, which has caused controversy in the field. Furthermore, while the reviewed studies demonstrate compelling correlations and suggest a potential role for irisin, particularly in CSF, as a marker associated with AD pathology, they fall short of validating it as a clinical biomarker. None of the included studies reported essential diagnostic accuracy metrics such as receiver operating characteristic (ROC) curves, area under the curve (AUC), sensitivity, specificity, or predictive values in independent cohorts. Claims of a “closer association” with Aβ are based on comparative correlation or regression analyses within studies, not on superior diagnostic performance. Therefore, irisin should currently be considered a candidate biomarker of significant mechanistic interest, not a bona fide diagnostic or prognostic tool. Future research must move beyond association to conduct rigorous diagnostic test accuracy studies in well-characterized, prospective cohorts to determine if irisin measurement has the necessary sensitivity, specificity, and additive value to be incorporated into clinical or research biomarker panels. A major limitation in irisin research is the lack of standardized measurement methods. Studies use varied assays (different ELISAs, mass spectrometry) with inconsistent antibodies and standards, leading to poor comparability and reproducibility of irisin levels across studies. This fundamental methodological issue complicates the interpretation of both clinical associations and preclinical findings, underscoring the need for a validated gold-standard assay to advance the field. The use of standardized, validated assays is paramount for future research and clinical application. Furthermore, an important and emerging consideration is the role of biological sex. Preclinical and clinical data suggest that irisin’s effects may be sexually dimorphic. For instance, in a tauopathy model, irisin treatment reduced pathological tau phosphorylation and neuroinflammation selectively in female mice [14]. Similarly, a clinical study reported that the correlation between reduced cerebrospinal fluid irisin levels and core Alzheimer’s disease biomarkers was more pronounced in women [20]. These findings underscore that sex is not merely a confounding variable but may influence the underlying pathophysiology, irisin’s mechanism of action, and therapeutic response. Future studies must be prospectively designed to explicitly account for and investigate these sex-specific effects. The optimal therapeutic window, dosing regimen, and delivery method (e.g., peripheral injection vs. central delivery or gene therapy) for irisin require thorough investigation. Finally, while its ability to cross the blood–brain barrier (BBB) is well-established [12,17], strategies to enhance its central delivery or prolong its half-life could improve therapeutic efficacy.

The findings of this review should be interpreted in light of the moderate risk of bias observed in some observational and preclinical studies, particularly related to residual confounding and incomplete reporting of methodological safeguards.

## 5. Conclusions

In conclusion, the evidence compiled in this review, supported by preclinical models, firmly establishes that irisin, a critical mediator of exercise, exerts neuroprotective effects across diverse neurological conditions via anti-inflammatory and direct neuronal protective mechanisms. Supportive clinical observations associate altered irisin levels with disease severity; however, the current human evidence remains largely associative. Thus, while irisin represents a compelling therapeutic candidate, its clinical efficacy awaits validation in future interventional trials. While further research is needed to standardize its measurement, understand its pharmacokinetics, clarify sex-dependent efficacy, and validate its efficacy in large-scale human trials, the robust preclinical evidence and consistent clinical associations summarized here establish irisin as a compelling candidate worthy of future translational research. However, its efficacy and safety as a therapeutic intervention in humans remain speculative and must be rigorously evaluated in future clinical trials.

## Figures and Tables

**Table 1:** Summary of Pre-Clinical Studies Investigating the Neuroprotective Effects of Irisin in Neurological and Neurodegenerative Conditions.

Table 1 Study Ref.	Study Type	Aims	Condition	Setting	Sample Size	Finding
Wang Y. et al., 2022 [5]	Experimental animal study	To investigate the impact of irisin treatment on neuroinflammation and neuronal apoptosis after intracerebral hemorrhage, and to explore the role of integrin αVβ5/AMPK signaling	Intracerebral hemorrhage (ICH)	Laboratory (intrastriatal injection model in mice)	Total mice used: 285 male C57BL/6 mice ICH group: 203 mice Excluded: 3 mice (no hematoma found) Mortality in ICH group: 10 out of 203 (4.93%)	Post-treatment with irisin improved neurological function, decreased brain edema, and reduced both neuroinflammation and neuronal apoptosis p<0.05
Kam T. et al., 2022 [7]	Pre-clinical (Animal and Cell Study)	To investigate if irisin prevents pathologic α-synuclein-induced neurodegeneration in a mouse model of Parkinson’s disease.	Parkinson’s Disease (PD)	Laboratory (in vivo mouse model and in vitro primary cortical neuron cultures)	Animal model (n = 12–13 in each group) and cell culture study (n = 3–4 per experiment)	Irisin reduced pathologic α-syn by enhancing endolysosomal degradation of pathologic α-syn p<0.05
Zhang X. et al., 2023 [4]	Pre-clinical (Animal/Cell Study)	To investigate the protective effects of irisin on Parkinson’s disease (PD) models and its mechanism of action.	Parkinson’s Disease (PD)	Laboratory (in vitro and in vivo models) and Clinical (PD patients) Shanghai Tongji Hospital, Tongji University School of Medicine e	Preclinical in vivo: 38 mice total. Preclinical in vitro: Minimum of 3 independent replicates per experiment.	Irisin exerts neuroprotective effects in Parkinson’s models by activating Akt/ERK signaling to improve mitochondrial function, reduce apoptosis/oxidative stress, and preserve motor and neuronal integrity, supporting its potential as a PD therapy, *p* < 0.05
Zhu, et al., 2025 [8]	Pre-clinical (In Vitro and In Vivo)	To investigate if irisin promotes autophagy and attenuates NLRP3 inflammasome activation in Parkinson’s disease models.	Parkinson’s Disease (PD)	Laboratory (Cell culture: BV2, SH-SY5Y; Animal model: C57BL/6 mice)	In Vitro: Not explicitly stated In Vivo: n = 6 mice per group	The findings suggest that irisin may offer neuroprotection against α-synuclein pathology in Parkinson’s disease, indicating its potential as a promising therapeutic target for both prevention and treatment. *p* < 0.05
Li N et al., 2025 [9]	Pre-clinical (Animal Study)	To investigate how exercise affects MPTP-induced PD pathology, focusing on the Irisin/AMPK/S IRT1 pathway, mitochondrial function, and apoptosis.	Parkinson’s Disease (PD)	Laboratory (Animal model)	32 male C57BL/6J mice (8 control, 24 MPTP-induced PD model)	Exercise elevated nigral irisin, *p*-AMPK, and SIRT1, which were reversed by Cyclo RGDyk, blocking AMPK/SIRT1 pathway activation and eroding mitochondrial benefits and anti-apoptotic effects. *p* < 0.05
Zarbakhsh S. et al., 2019 [10]	Pre-clinical (Animal Study)	To evaluate whether co-treatment with Irisin and bone marrow stem cells (BMSCs) can protect dopaminergic neurons in a Parkinson’s disease model	Parkinson’s Disease (PD)	Laboratory (Animal model)	35 adult male Wistar rats (divided into 5 groups of n = 7	Co-treatment with irisin and BMSCs protects dopaminergic neurons from degeneration and apoptosis following MPTP administration
Lourenco, et al., 2019 [11]	Pre-clinical (Animal/Cell Study)	To investigate the role of exercise-induced FNDC5/irisin in synaptic plasticity and memory in Alzheimer’s disease models.	Alzheimer’s Disease (AD)	Laboratory (in vitro, in vivo models) and Clinical (human post-mortem brain tissue, CSF)	Human: 11 controls, 7 early AD, 7 late AD (brain); 26 controls, 14 MCI, 14 AD, 13 LBD (CSF) Animal: Various group sizes (e.g., n = 4–12 mice/group)	Brain-specific FNDC5/irisin knockdown impaired synaptic plasticity and memory in mice, while irisin restoration through adenoviral overexpression or exercise rescued these deficits in Alzheimer’s disease models
Islam, et al., (2021) [12]	Preclinical Animal Study	To determine if the exercise-induced hormone irisin is the critical mediator of exercise’s cognitive benefits and to evaluate its therapeutic potential for Alzheimer’s disease (AD).	Cognitive Function, Aging, Alzheimer’s Disease	Laboratory (in vivo & in vitro)	Mice: Global FNDC5 KO (FSKO), WT, APP/PS1, 5xFAD models. Sample sizes vary per experiment (e.g., n = 5–15/group).	Peripheral delivery of irisin (via AAV in liver) elevated central irisin levels, crossed the BBB, and improved cognitive function and reduced neuroinflammation in two AD mouse models (APP/PS1 and 5xFAD)
Zhang, et al., 2025 [13]	Preclinical (In vivo and In vitro)	To investigate the protective effects and mechanisms of irisin on LPS-induced inflammatory cognitive impairment.	Lipopolysaccharide (LPS)-induced neuroinflammation and cognitive impairment.	Laboratory setting (Animal facility and cell culture lab) Department of Neurology, Hospital of Hainan Medical University, Haikou, China	In vivo: 36 mice (n = 12/group) In vitro: BV2 and PC12 cell lines (n = 3–6/group, repeated 3 times)	Irisin exerted neuroprotection in LPS-treated mice by improving cognition and attenuating hippocampal injury. These effects were mediated by shifting microglial polarization from M1 to M2, suppressing the NLRP3 inflammasome, reducing cytokine release, and shielding neurons from microglia-driven toxicity and apoptosis
Lourenco, et al., 2022 [3]	In vitro (primary rat hippocampal neurons)	To investigate the neuroprotective signaling pathways stimulated by irisin in hippocampal neurons.	Alzheimer’s Disease (AD) pathology, Oxidative stress	Laboratory (cell culture) and Analysis of human RNAseq dataset (Aging, Dementia and TBI Study)	n = 3–7 independent culture experiments per assay.	Results show that irisin triggers protective responses in hippocampal neurons, supporting the potential of irisin signaling as a beneficial strategy for Alzheimer’s disease.
Bretland, et al., 2021 [14]	In vivo (animal model)	To determine if irisin could prevent the emergence of early tau pathology and neuroinflammation in a pre-symptomatic tauopathy model	Alzheimer’s Disease (tauopathy)	Northeast Ohio Medical University, Rootstown, OH, USA.	30 mice for primary analysis (16 transgenic htau, 14 non-transgenic C57BL/6J controls)	Results suggest therapeutic potential for irisin in reducing early tau pathology and neuroinflammation, with efficacy observed only in females
Zhang, et al., 2024 [15]	In vivo (mouse model) and In vitro (cell line)	To investigate the neuroprotective effects of irisin and its mechanism of action via the integrin αVβ5/AMPK/autophagy pathway in microglia.	Acute Ocular Hypertension (AOH)- induced retinal injury (model for acute glaucoma) and LPS-induced microglial inflammation.	Laboratory (Tongji Hospital, China)	Male C57BL/6, WT, and FNDC5-/-mice (6–8 weeks old). Group sizes ranged from n = 3 to n = 7 per experiment. In vitro: BV2 microglial cell line.	Irisin attenuates AOH-induced neuroinflammation and retinal ganglion cell (RGC) death. ultimately providing a neuroprotective effect.
Ozdemir-Kumral, et al., 2024 [16]	In vivo (rat model)	To evaluate the neuroprotective effects of centrally administered irisin and acute exhausting exercise against oxidative brain injury and memory dysfunction due to a pentylenetetrazole (PTZ)-induced single seizure.	Pentylenetetrazole (PTZ)-induced epileptic seizure	Laboratory (Marmara University, Türkiye)	48 female Sprague-Dawley rats (230–280 g, 12 weeks old). 8 rats per experimental group.	Both central administration of irisin and acute exhaustive exercise before seizure induction conferred neuroprotection by delaying seizure onset, restoring the glutamate/GABA balance, reducing oxidative stress, preserving antioxidant defenses, minimizing neuronal damage, and improving seizure-related memory deficits, with exercise additionally enhancing cerebral BDNF Expression.
Wang, et al., 2025 [17]	Preclinical (Mice)	To investigate the role of irisin in preventing Postoperative Cognitive Dysfunction (POCD) and its mechanism of action via microglial reprogramming.	Postoperative Cognitive Dysfunction (POCD), Neuroinflammation, Dementia	Laboratory (Peking University) and Hospital (Peking University First Hospital)	Mice: Group sizes typically n = 5–8 for molecular, n = 8–13 for behavioral tests.	Irisin pretreatment prevented surgery- or LPS-induced neuroinflammation, neuronal hyperexcitability, synaptic loss, and cognitive impairment, while prophylactic administration reduced early postoperative cognitive dysfunction and alleviated anesthesia-induced hypothermia.

**Table 2:** Clinical Observational Studies Investigating Irisin Levels and Their Associations with Neurological and Neurodegenerative Disorders.

Table 2 Study Ref.	Study Type	Aims	Condition	Setting	Sample Size	Finding
Wu H. et al., 2019 [18]	Prospective observational clinical study (3-month follow-up)	To determine serum irisin levels and investigate their associations with functional outcomes in first-ever acute ischemic stroke (AIS) patients	Acute ischemic stroke (AIS)	Department of Emergency, Zhongnan Hospital of Wuhan University, Wuhan, China.	324 AIS patients	A low serum irisin level is a predictor of poor early functional outcome in ischemic stroke patients
Tu W.J. et al., 2018 [19]	Prospective observational cohort study (6-month follow-up)	To determine serum irisin levels and investigate their associations with post-stroke depression (PSD) after first-ever acute ischemic stroke	Post-stroke depression (PSD) in acute ischemic stroke (AIS) patients	Three stroke centers in China	1205 AIS patients	The data suggested that reduced serum levels of irisin were powerful biological markers for the risk of developing PSD even after adjustment by variables
Chen Y. et al., 2022 [20]	Randomized Controlled Trial (RCT)	To observe the effect of staged acupuncture on serum irisin level, neurological deficit, balance ability, and spasticity in patients with ischemic stroke.	Ischemic Stroke	Clinical (likely a hospital or research center, as patients and healthy subjects were recruited)	90 total (60 patients divided into two groups of 30, plus 30 healthy subjects in a normal group)	The staged acupuncture could increase the serum irisin level, improve the neurological function, balance ability and spasticity in patients with ischemic stroke
Zhang X. et al., 2023 [4]	Human Clinical Observation	To investigate the protective effects of irisin on Parkinson’s disease (PD) models and its mechanism of action.	Parkinson’s Disease (PD)	Shanghai Tongji Hospital, Tongji University School of Medicine	23 patients	Peripherally delivered irisin might be a promising candidate for therapeutic targeting of PD.
Shi, et al., 2024 [21]	Case–control study	To investigate the relationship of irisin with disease severity and dopamine uptake in Parkinson’s disease patients.	Parkinson’s Disease (PD)	Henan Provincial People’s Hospital, Zhengzhou, China	100 PD patients and 70 healthy controls	Plasma irisin levels were reduced in Parkinson’s disease, declined with disease progression, inversely correlated with α-synuclein and motor severity (UPDRS-III), positively correlated with cognitive function (MoCA), and were associated with greater striatal dopamine uptake, particularly contralateral to the affected limb
Lourenco, et al., 2019 [11]	Human Clinical Observation	To investigate the role of exercise-induced FNDC5/irisin in synaptic plasticity and memory in Alzheimer’s disease models.	Alzheimer’s Disease (AD)	Human post-mortem brain tissue, CSF)	CSF and plasma cohort: Controls: n = 26 MCI: n = 14 AD: n = 14 (plasma AD n = 13 in some analyses) LBD: n = 13–14	FNDC5/irisin is significantly reduced in the AD brain and CSF, while plasma levels remain largely unchanged, resulting in a decreased CSF/plasma irisin ratio; these findings indicate a CNS-specific dysregulation of irisin that is disrupted in Alzheimer’s disease and Lewy body dementia
Pignataro P. et al., 2025 [22]	Clinical Cohort Study	To investigate the association of cerebrospinal fluid (CSF) and serum irisin levels with multidomain cognition in a biologically defined cohort of patients with Alzheimer’s disease (AD) pathology.	Alzheimer’s Disease (AD), Mild Cognitive Impairment (MCI), Subjective Memory Complaint (SMC)	University of Bari “A. Moro”, Bari, Italy	146 subjects total: AD (n = 82), MCI (n = 44), SMC (n = 20)	CSF and irisin levels significantly correlated with global cognitive efficiency (MMSE) and multiple specific cognitive domains (memory, executive functions, attention, visuospatial abilities, language).
Dicarlo, et al. (2024) [23]	Clinical Cohort Study	To study CSF and plasma irisin levels, their correlation with AD biomarkers (Aβ, tau) and clinical scores (CDR-SOB), and investigate sex differences.	Alzheimer’s Disease (AD), Mild Cognitive Impairment (MCI), Subjective Memory Complaint (SMC)	University of Bari “A. Moro”, Bari, Italy.	146 subjects total: AD Dementia (n = 82), MCI (n = 44), SMC (n = 20)	Findings suggest CSF irisin is a potential biomarker for AD progression, with a more pronounced role in female pathophysiology.
Lourenco, et al. (2020) [24]	Clinical Cohort Study	To investigate whether cerebrospinal fluid (CSF) irisin levels correlate with AD biomarkers (Aβ, tau), brain-derived neurotrophic factor (BDNF), and cognitive performance in humans	Alzheimer’s Disease (AD), Non-Demented Controls (NDC)	Memory clinic at D’Or Institute of Research and Education (IDOR) in Rio de Janeiro, Brazil	39 subjects total: AD (n = 14), Non-Demented Controls (NDC; n = 25)	Findings suggest that reduced CSF irisin and BDNF in AD are more closely related to amyloid pathology than to general neurodegeneration (as measured by t-tau).
Lourenco et al., 2022 [3]	Human post-mortem data analysis	To investigate the neuroprotective signaling pathways stimulated by irisin in hippocampal neurons.	Alzheimer’s Disease (AD) pathology, Oxidative stress	Laboratory (cell culture) and Analysis of human RNAseq dataset (Aging, Dementia and TBI Study)	Subjects > 77 years old (n is not reported).	Hippocampal FNDC5/irisin expression shows an age-related decline and is inversely associated with Alzheimer’s disease neuropathology, particularly tau burden, indicating disrupted brain irisin signaling in aging and AD.
Huang X. et al., 2024 [25]	Cross-sectional study	To examine differences in plasma biomarkers between people with MCI and cognitively normal individuals, and explore their relations with cognitive performance.	Mild Cognitive Impairment (MCI)	Shanghai Jiao Tong University, Shanghai, China	250 older adults (124 with MCI, 126 cognitively normal)	The plasma BDNF/irisin ratio may be a reliable biomarker for reflecting MCI odds and cognitive performance.
Wang, et al., 2025 [17]	Observational, cross-sectional case–control study	To investigate the role of irisin in preventing Postoperative Cognitive Dysfunction (POCD) and its mechanism of action via microglial reprogramming.	Postoperative Cognitive Dysfunction (POCD), Neuroinflammation, Dementia	Peking University First Hospital	Human: 37 patients (18 Control, 19 Dementia)	In elderly patients (>70 years), lower preoperative serum irisin levels were associated with an increased risk of long-term postoperative dementia, showing a ~31% reduction compared with cognitively normal controls and a negative correlation with age in affected individuals

## Data Availability

The original contributions presented in this study are included in the article/Supplementary Material. Further inquiries can be directed to the corresponding author.

## Supplementary Materials

The following supporting information can be downloaded at: https://www.mdpi.com/article/10.3390/neurolint18010015/s1, Table S1: Risk of Bias Assessment of Clinical Studies Using the Newcastle–Ottawa Scale (NOS); Table S2: Risk of Bias Assessment of Randomized Controlled Trial Using Cochrane RoB 2; Table S3: Risk of Bias Assessment of Preclinical Animal Studies Using SYRCLE; Table S4: Risk of Bias Assessment of In Vitro Studies Using OHAT-Adapted Domains; Supplementary File S1: PRISMA 2020 for Abstracts Checklist; Supplementary File S2: PRISMA 2020 Checklist [26].

## Abbreviations

The following abbreviations are used in this manuscript:

PD	Parkinson’s disease
AD	Alzheimer’s disease
FNDC5	Fibronectin type III domain-containing protein 5
BDNF	Brain-derived neurotrophic factor

## References

[1] Alzoughool F., Al-Zghoul M.B., Ghanim B.Y., Gollob M., Idkaidek N., Qinna N.A. (2022). The Role of Interventional Irisin on Heart Molecular Physiology. Pharmaceuticals.

[2] Wrann C.D., White J.P., Salogiannnis J., Laznik-Bogoslavski D., Wu J., Ma D., Lin J.D., Greenberg M.E., Spiegelman B.M. (2013). Exercise induces hippocampal BDNF through a PGC-1α/FNDC5 pathway. Cell Metab..

[3] Lourenco M.V., de Freitas G.B., Raony Í., Ferreira S.T., De Felice F.G. (2022). Irisin stimulates protective signaling pathways in rat hippocampal neurons. Front. Cell. Neurosci..

[4] Zhang X., Xu S., Hu Y., Liu Q., Liu C., Chai H., Luo Y., Jin L., Li S. (2023). Irisin exhibits neuroprotection by preventing mitochondrial damage in Parkinson’s disease. NPJ Park. Dis..

[5] Wang Y., Tian M., Tan J., Pei X., Lu C., Xin Y., Deng S., Zhao F., Gao Y., Gong Y. (2022). Irisin ameliorates neuroinflammation and neuronal apoptosis through integrin αVβ5/AMPK signaling pathway after intracerebral hemorrhage in mice. J. Neuroinflamm..

[6] Liu Y., Zhu C., Guo J., Chen Y., Meng C. (2020). The Neuroprotective Effect of Irisin in Ischemic Stroke. Front. Aging Neurosci..

[7] Kam T.-I., Park H., Chou S.-C., Van Vranken J.G., Mittenbühler M.J., Kim H., A M., Choi Y.R., Biswas D., Wang J. (2022). Amelioration of pathologic α-synuclein-induced Parkinson’s disease by irisin. Proc. Natl. Acad. Sci. USA.

[8] Zhu M., Peng Q., Li S., Zhang G., Zhang Z. (2025). Irisin promotes autophagy and attenuates NLRP3 inflammasome activation in Parkinson’s disease. Int. Immunopharmacol..

[9] Li N., Wang B., Wang Y., Tian X., Lin J., Sun X., Sun Y., Zhang X., Xu H., Li M. (2025). Exercise Ameliorates Dysregulated Mitochondrial Fission, Mitochondrial Respiration, and Neuronal Apoptosis in Parkinson’s Disease Mice via the Irisin/AMPK/SIRT1 Pathway. Mol. Neurobiol..

[10] Zarbakhsh S., Safari M., Aldaghi M.R., Sameni H., Ghahari L., Lagmouj Y.K., Jaberi K.R., Parsaie H. (2019). Irisin protects the substantia nigra dopaminergic neurons in the rat model of Parkinson’s disease. Iran J. Basic Med. Sci..

[11] Lourenco M.V., Frozza R.L., De Freitas G.B., Zhang H., Kincheski G.C., Ribeiro F.C., Gonçalves R.A., Clarke J.R., Beckman D., Staniszewski A. (2019). Exercise-linked FNDC5/irisin rescues synaptic plasticity and memory defects in Alzheimer’s models. Nat. Med..

[12] Islam M.R., Valaris S., Young M.F., Haley E.B., Luo R., Bond S.F., Mazuera S., Kitchen R.R., Caldarone B.J., Bettio L.E.B. (2021). Exercise hormone irisin is a critical regulator of cognitive function. Nat. Metab..

[13] Zhang D.-Q., Li G., Fu Z.-D., Huang Y.-S., Feng X.-L., Chen L.-J., Wang R., Zhao W.-J., Li Q. (2025). Irisin ameliorates cognitive impairment in a lipopolysaccharide-induced neuroinflammation mouse model by inhibiting the NLRP3 inflammasome pathway in microglia. Neuropharmacology.

[14] Bretland K.A., Lin L., Bretland K.M., Smith M.A., Fleming S.M., Dengler-Crish C.M. (2021). Irisin treatment lowers levels of phosphorylated tau in the hippocampus of pre-symptomatic female but not male htau mice. Neuropathol. Appl. Neurobiol..

[15] Zhang Q., Xiang S., Chen X., Rong Y., Huang L., Chen Z., Yao K., Chen W., Deng C., Wang J. (2024). Irisin attenuates acute glaucoma-induced neuroinflammation by activating microglia-integrin αVβ5/AMPK and promoting autophagy. Int. Immunopharmacol..

[16] Ozdemir-Kumral Z.N., Akgün T., Haşim C., Ulusoy E., Kalpakçıoğlu M.K., Yüksel M.F., Okumuş T., Us Z., Akakın D., Yüksel M. (2024). Intracerebroventricular administration of the exercise hormone irisin or acute strenuous exercise alleviates epileptic seizure-induced neuroinflammation and improves memory dysfunction in rats. BMC Neurosci..

[17] Wang J., Gao S., Fu S., Li Y., Su L., Li X., Wu G., Jiang J., Zhao Z., Yang C. (2025). Irisin reprograms microglia through activation of STAT6 and prevents cognitive dysfunction after surgery in mice. Brain Behav. Immun..

[18] Wu H., Guo P., Jin Z., Li X., Yang X., Tang C., Wang Y., Ke J. (2019). Serum levels of irisin predict short-term outcomes in ischemic stroke. Cytokine.

[19] Tu W.-J., Qiu H.-C., Liu Q., Li X., Zhao J.-Z., Zeng X. (2018). Decreased level of irisin, a skeletal muscle cell-derived myokine, is associated with post-stroke depression in the ischemic stroke population. J. Neuroinflamm..

[20] Chen Y., Du Z.-H., Chen H.-Y., Pan Y. (2022). Effect of staged acupuncture on serum irisin level and neurological rehabilitation in patients with ischemic stroke. Zhongguo Zhen Jiu.

[21] Shi X., Gu Q., Fu C., Ma J., Li D., Zheng J., Chen S., She Z., Qi X., Li X. (2024). Relationship of irisin with disease severity and dopamine uptake in Parkinson’s disease patients. NeuroImage Clin..

[22] Pignataro P., Dicarlo M., Zecca C., Urso D., Dell’ABate M.T., Vilella D., Borlizzi F., Zerlotin R., Oranger A., Colaianni G. (2025). Correlation Between Irisin and Cognitive Functions in Alzheimer Dementia. Ann. Clin. Transl. Neurol..

[23] Dicarlo M., Pignataro P., Zecca C., Dell’Abate M.T., Urso D., Gnoni V., Giugno A., Borlizzi F., Zerlotin R., Oranger A. (2024). Irisin Levels in Cerebrospinal Fluid Correlate with Biomarkers and Clinical Dementia Scores in Alzheimer Disease. Ann. Neurol..

[24] Lourenco M.V., Ribeiro F.C., Sudo F.K., Drummond C., Assunção N., Vanderborght B., Tovar-Moll F., Mattos P., De Felice F.G., Ferreira S.T. (2020). Cerebrospinal fluid irisin correlates with amyloid-β, BDNF, and cognition in Alzheimer’s disease. Alzheimer’s Dement..

[25] Huang X., Wang J., Zhang S., Zhao X., An R., Lan Y., Yi M., Wan Q. (2024). Plasma BDNF/Irisin Ratio Associates with Cognitive Function in Older People. J. Alzheimer’s Dis..

[26] Page M.J., McKenzie J.E., Bossuyt P.M., Boutron I., Hoffmann T.C., Mulrow C.D., Shamseer L., Tetzlaff J.M., Akl E.A., Brennan S.E. (2021). The PRISMA 2020 statement: An updated guideline for reporting systematic reviews. BMJ.

