# Supplementary material for: The Multifaceted Role of Irisin in Neurological Disorders: A Systematic Review Integrating Preclinical Evidence with Clinical Observations
Source: Neurol Int. 2026 Jan 9;18(1):15. doi: 10.3390/neurolint18010015 (PMC12844615; doi:10.3390/neurolint18010015)
Supplement: Supplementary file 1 [file neurolint-18-00015-s001.zip › neurolint-4024273-supplementary tableS1-4.pdf]

Supplementary Table 1 (S1): Risk of Bias Assessment of Clinical Studies Using the Newcastle–Ottawa Scale (NOS)

Study	Selection	Comparability	Outcome/Exposure	Overall Quality
Wu et al., 2019	★★★★	★★	★★★	High
Tu et al., 2018	★★★★	★★	★★★	High
Shi et al., 2024	★★★	★★	★★★	High
Pignataro et al., 2025	★★★★	★★	★★★	High
Dicarlo et al., 2024	★★★★	★★	★★★	High
Huang et al., 2024	★★★	★	★★★	Moderate
Wang et al., 2025	★★★	★	★★★	Moderate

Note: The NOS evaluates methodological quality across three domains: Selection (maximum 4 stars), Comparability (maximum 2 stars), and Outcome/Exposure (maximum 3 stars). Overall study quality was generally classified as High (≥7 stars), Moderate (5–6 stars), or Low (<5 stars). To ensure a conservative interpretation, studies receiving ≤1 star in the Comparability domain were downgraded to Moderate quality even if the total score reached 7 stars, reflecting potential residual confounding inherent to observational designs.

Supplementary Table 2 (S2): Risk of Bias Assessment of Randomized Controlled Trial Using Cochrane RoB 2

Study	Randomization	Deviations	Missing Data	Outcome Measurement	Reporting	Overall Risk
Chen et al., 2022	Low	Low	Low	Some concerns	Low	Low

Note: RoB-2 tool evaluates methodological quality across five domains: randomization process, deviations from intended interventions, missing outcome data, measurement of outcomes, and selection of the reported result. Overall risk of bias was judged as Low when all domains were rated low risk, Some concerns when at least one domain raised some concerns without high risk, and High when one or more domains were rated high risk.

Supplementary Table 3 (S3): Risk of Bias Assessment of Preclinical Animal Studies Using SYRCLE

Study	Selection Bias	Performance Bias	Detection Bias	Attrition Bias	Reporting Bias	Overall Risk
Wang et al., 2022	Unclear	Unclear	Low	Low	Low	Moderate
Kam et al., 2022	Unclear	Unclear	Unclear	Low	Low	Moderate
Li et al., 2025	Unclear	Unclear	Unclear	Low	Low	Moderate
Islam et al., 2021	Unclear	Unclear	Low	Low	Low	Moderate
Bretland et al., 2021	Low	Low	Low	Low	Low	Low
Note: The SYRCLE tool evaluates selection, performance, detection, attrition, and reporting biases in animal studies. Each domain was rated as Low, High, or Unclear risk of bias. Overall risk of bias was considered Low when most domains were rated low risk, Moderate when multiple domains were rated unclear without high-risk judgments, and High when one or more domains were rated high risk.						

Supplementary Table 4 (S4): Risk of Bias Assessment of In Vitro Studies Using OHAT-Adapted Domains

Study	Exposure Characterization	Outcome Assessment	Confounding	Reporting Transparency	Overall Risk
Lourenco et al., 2022	Low	Low	Low	Low	Low
Zhang et al., 2024	Low	Low	Low	Low	Low
Note: In vitro studies were assessed using adapted OHAT risk-of-bias domains, including exposure characterization, outcome assessment, confounding control, and reporting transparency. Each domain was rated as Low risk or Some concerns. Overall risk of bias was judged as Low when all domains were rated low risk, and Moderate when one or more domains raised some concerns.					
